# Factors that drive team participation in surgical safety checks: a prospective study

**DOI:** 10.1186/s13037-015-0090-5

**Published:** 2016-01-20

**Authors:** Brigid M. Gillespie, Teresa K. Withers, Joanne Lavin, Therese Gardiner, Andrea P. Marshall

**Affiliations:** NHMRC Centre for Research Excellence in Nursing (NCREN), Centre for Health Practice Innovation (HPI), Menzies Health Institute Qld (MHIQ), Griffith University, Parklands Drive, Gold Coast Campus, Gold Coast, QLD 4222 Australia; Gold Coast University Hospital, Gold Coast Hospital and Health Service, 1 Hospital Boulevard, Southport, QLD 4215 Australia; School of Nursing and Midwifery, Griffith University, Gold Coast Campus, Nathan, QLD 4222 Australia

## Abstract

**Background:**

Team-based group communications using checklists are widely advocated to achieve shared understandings and improve patient safety. Despite the positive effect checklists have on collaborations and reduced postoperative complications, their use has not been straightforward. Previous research has described contextual factors that impact on the implementation of checklists, however there is limited understanding of the issues that impede team participation in checklist use in surgery. The aim of this prospective study was to identify and describe factors that drive team participation in safety checks in surgery.

**Methods:**

We observed ten surgical teams and conducted 33 semi-structured interviews with 70 participants from nursing, surgery and anaesthetics, and the community. Constant comparative methods were used to analyse textual data derived from field notes and interviews. Observational and interview data were collected during 2014–15.

**Results:**

Analysis of the textual data generated from the field notes and interviews revealed the extent to which members of the surgical team participated in using the surgical safety checklist during each phase of patient care. These three categories included: ‘using the checklist’; ‘working independently’; and, ‘communicating checks with others’. The phases in the checking process most vulnerable to information loss or omission were *sign in* and *sign out*.

**Conclusions:**

Team participation in safety checks depends on a convergence of intertwined factors; namely, *team attributes, communication strategies* and *checking processes.* A whole-of-team approach to participation in surgical safety checks is far more complex when considering the factors that drive participation. Strategies to increase participation in safety checks need to target professional communication practices and work processes such as workflow which curtail team members’ ability to participate.

## Background

The 1999 *Institute of Medicine* (IOM) report, “To err is human” estimated that between 44,000 and 98,000 patients die each year as a result of medical errors in the United States [1]. Other studies suggest that the incidence rate lies somewhere between 2.9 % (499/17,192) [2] and 16.6 % (2,353/14,179) [3]. Of concern is that nearly 45 % (7,712/17,192) of adverse events (AE) occur in surgery, with a median of 43.5 % (interquartile range 39.4 to 49.6 %) of these considered as avoidable [4]. The incidence of major complications as a result of surgery is estimated to be between 23.3 % (272/1,177) [5] and 77 % (112/146) [6] of inpatient surgical procedures. In an effort to reduce AE and complication rates in surgery, the World Health Organisation (WHO) launched the *Safe Surgery Saves Lives* campaign in 2008 [7], advocating the use of checklists worldwide.

Since its introduction in over 122 countries, the WHO Surgical Safety Checklist (SSC) has been the focus of many published studies [8–13], including several meta-analyses [14–16], suggesting associated reductions in patient mortality and postoperative complication rates when the SSC is used. The SSC is intended as an ‘aid memoir’ for including key information or actions that may otherwise be overlooked or forgotten, thus ensuring timely and consistent communication among surgical team members [2, 3]. The 19 item checklist has three time points that reflect the take-off, cruise and landing phases of the aviation industry [7, 17]. Before anaesthesia induction *sign in*, the surgical team confirms patient identity, surgical site, anaesthetic concerns and estimated blood loss. Prior to skin incision, the entire team performs *time out*, a surgical ‘pause’ where anticipated critical events are reviewed, sterility and antibiotic status is confirmed, and imaging is verified and displayed. Before the patient leaves the operating room (OR), the *sign out* phase includes confirmation of the surgical count, procedure name, handling of tissue specimens, and ensuring equipment issues are addressed.

Despite the benefits associated with the SSC, recent research suggests universal implementation and continued use in the clinical environment is often inconsistent, and very much driven by the context [14, 18–20]. Enablers to SSC use include physician engagement and leadership [18], tailoring and customizing the checklist to the local context [18, 20], and creating opportunities for stakeholder reflection on checklist implementation and modification, giving them greater ownership of the process [18]. Common identified barriers to sustained use of checklists in surgery include haphazard introduction [19, 20], perceived duplication or redundancy of information [19], professional identification [18, 20, 21] and team culture [18, 19]. The barriers and enablers to implementation identified across these studies predominantly encompass organizational and individual factors.

However, findings from previous studies in this area [22–25] suggest that checklist implementation does not always equate to compliance. In fact, two studies [24, 26] found that despite 100 % documented completion of the *sign in* phase of the checklist, most of the individual items were either not addressed as intended, or not addressed at all. Variation in compliance rates across studies has been noted in many of the prospective studies conducted in this area [10, 22, 27, 28]. Failure to consistently review all checklist items may increase patients’ risk of preventable ‘never events’ such as wrong site/side surgery or retained foreign bodies [29]. Consequently, identifying issues that act as barriers and enablers to checklist implementation may inform the development of knowledge translation strategies designed to increase item usage and overall uptake.

## Methods

This prospective study is part of a larger mixed methods knowledge translation study where the aim was to evaluate the feasibility and acceptability of a multifaceted behaviour change intervention on SSC use. The aim of this prospective study was to identify and describe factors that act as barriers or enablers to team participation in SSC checks.

### Setting and sample

The study setting was a 550 bed Australian tertiary centre, specializing in paediatrics, orthopaedics, vascular, cardiac, obstetrics/gynaecology, general, urology, ENT, plastics, neuro, ophthalmology, trauma, and thoracic surgeries. At the time of data collection, the perioperative department had 16/20 ORs commissioned and was staffed by over 300 nurses, physicians and ancillary staff. At this facility, approximately 20,000 surgical procedures are performed each year.

The selection of surgical procedures and participants was purposive and clinical participants were drawn from anaesthetics, surgery, and nursing, working across 12 surgical specialities. Healthcare consumers were selected based on their ability to reflect on their previous experiences as a surgical patient in the context of using checklists as a safety tool to prevent harm. Maximum variation in sampling [30] was used to achieve diversity in participants’ experiences and perceptions of team communications using the SSC, based on their professional and personal backgrounds.

### Data collection

Observational and interview data were collected over 8 months during 2014–15. Data collection methods included structured observations, field notes, and individual and group interviews. The observational audit was performed by a member of the research team who was an experienced OR nurse, and trained in observational methods. The researcher used a standardized data collection tool in a tabular format and collected data on checklist item usage rates and team member presence and participation across the three phases (i.e., *sign in, timeout, sign out*) of the checking process. ‘Participation’ in checking processes was considered in relation to behaviours such as active listening, asking questions and/or answering queries, and passing on clinical information when relevant, to others. Checklist items were deemed incomplete if an anaesthetist, surgeon, and nurse did not confirm patient, site, procedure or any other relevant checks of each phase of the checklist.

Interviews were semi structured and were used to elicit clinician participants’ perspectives about the barriers and enablers of SSC implementation and use in practice. Interviews with the healthcare consumers were conducted to establish consumers’ expectations as recipients of surgical care. Semi structured interviews were digitally recorded and transcribed per verbatim. Prior to each interview, all participants completed a demographic questionnaire of characteristics (i.e., age, gender, education, clinical role) to contextualize the findings and describe the group. Participants were interviewed either individually or in groups, depending on their availability. Interviews were conducted at the participant’s convenience, away from the clinical environment. Group interviews were discipline-specific and were conducted with staff who belonged to the same staff category to diffuse potential status differentials [31]. Interview questions were based on Mitchie et al. [32]*.* ‘Theoretical Domains Framework’ (TDF) which targets aspects of individual behaviour relative to *knowledge, skills, professional role/identity, self-efficacy, outcome expectations, motivation/goals, environmental context/resources*, and *social norms*. For instance, to determine whether *knowledge* was a barrier or an enabler, we asked clinician participants, “What is your understanding of the purpose of the SSC?”, and, “Can you describe how you do the SSC?” Examples of questions asked of healthcare consumers included, “Do you believe that using a checklist can help reduce mistakes or errors?”, and, “Do you think that the checklist could improve communication among staff members in surgery?” The lead author performed all interviews and the number of interviews was determined by the point at which data saturation occurred.

### Ethical considerations

Approvals were given by the hospital and university Human Research Ethics Committees. Participants were given an invitational letter detailing study information and were required to sign a consent form. They were advised of their right to confidentiality and anonymity and to withdraw from the study at any time.

### Data analysis and rigour

From the textual analysis of field notes and interview transcripts, analysis occurred in an iterative manner to develop the subcategories and categories. The process of category development involved breaking down, comparing, and conceptualizing data to enable recognition of emerging patterns to identify major categories and their subcategories [33–35]. The extracted categories and their corresponding explanatory data were cross-checked among researchers to ensure consensus. In data analysis, investigator triangulation was used as all members of our interdisciplinary research team contributed to the findings using varied perspectives and discipline-specific expertise [36]. In keeping with an integrated knowledge translation approach [37], we presented the findings to our participants to verify their authenticity and accuracy at each stage of the analysis. Data saturation at the point of analysis was evident when no new information or categories emerged.

The hallmarks of rigour concerning credibility, auditability, transferability and triangulation [30, 33, 36] have been considered in this study. Credibility was evident in relation to giving participants opportunity to corroborate the authenticity of thematic findings with their experiences. An audit trail, supported by memos, linked to pieces of data confirmed the categories and subcategories. Transferability of findings to other similar settings was achieved through using a wide variety of participants that included clinicians and health consumers. We used a triangulated approach on two levels: First, we used different data sources enabling cross checking, confirmation and completeness of the data [36]. Second, the interdisciplinary approach we used enabled a comprehensive examination of the data set, reducing the potential for bias that is inherent in any study.

## Results

Approximately 35 h of field observations were conducted with 80 nurses, anaesthetists and surgeons working across 10 surgical teams in paediatrics, obstetrics/gynaecology, general surgery, and urology. The length of surgery ranged from 60 to 120 min (median 88 min, IQR 104 min). In total, 33 interviews were conducted with 70 participants in 10 focus groups and 23 individual interviews from nursing, medicine and healthcare consumers (Table 1). A total of 55 nurses, 11 physicians, and 4 healthcare consumers were interviewed. Interviews lasted between 12 and 50 min. Most participants were female (77 %). Participants’ ages ranged from 22 to 76 years, with a mean age of 43.5 years (*SD*13.0 years). Analysis of the observational audit, field notes and interviews informed the development of three categories and their subcategories (Table 2). A description of these categories follows.

**Table 1:** Number of participants (*n* = 70) interviewed and the method of interview (*n* = 33)

Number of participants	Method of interview	Role or 7specialty
10 Registered Nurses	Individual	• 3 Anaesthetics • 5 Scrub/scout • 2 PACU
9 Physicians	Individual	• 5 Anaesthetists • 4 Surgeons
4 Surgical Patients	Individual	• 4 Healthcare Consumers
45 Registered Nurses	9 Groups	• 9 Anaesthetics • 18 Scrub/scout • 11 PACU • 7 Management/Education
2 Physicians	Group	• 2 Anaesthetists

*Abbreviations: PACU* Post-anaesthetic recovery unit

**Table 2:** Categories, subcategories and examples of supporting verbatim

Category	Subcategory	Examples of supporting Verbatim
Using the checklist	Picking up problems	• *The consent said the patient was having his operation on the left hand. It was an emergency operation late at night, the anaesthetist had taken the patient into theatre without talking to the staff and proceeded to give the patient sedation. Then the nursing staff looked at it all and went “nup”. This patient has got the wrong consent!* (Int 10, RN) • *And everything should be about layers. So you know the front door should have a layer. The anaesthetic bay should have a layer. The final time out is here. Where it’s most important that everybody check things before any skin is cut….* (Int 28, Surgeon)
	Prompting, providing reminders	• *And you’ve got reminders, whether it’s the specimen, or the diathermy pad. You know, just so that you’ve completely followed through. It’s a good starting point, as a handover to the recovery phase. (Int 1, RN)* • *I guess effectively it’s almost a tick and flick but occasionally something pops up in the process of ticking and flicking there to remind you about something.* (Int 26, Anaesthetist)
	Tick-n-flick, going through the motions	• *The last bit is not done very well, the signout. That’s a tick tick tick thing*. (Int 4, 5 RNs) • *If you are not careful about what you are doing, the patient will get checked in and if all the boxes are ticked and nobody actually talks to the patient and confirms with the patient while they are awake that the surgeon knows what they are doing, it doesn’t prevent anything.* (Int 18, surgeon)
	Checking and rechecking	• *So I always check, are you happy with consent prior to bringing the patient in or giving sedation. I never do anything without one of the nurses. I don’t care if another doctor and the surgeon says consent is there. Have the nurses seen consent? Because I think they are much better at checklists than we are.* (Int 8, Anaesthetist) • *……you’re just basically double-checking everything, three, four, five million times, so it can slow down your day then because you want to make sure that the patient comes first and their safety is imperative in our role….* (Int 24, RN)
	Modifying and adapting	• *We’ve added in cardiac, for example, to make sure that we got, we say “pacing wires”, so they are not meant to be sent up.* (Int 20, RN cardiac) • *I might have pinned the head in the wrong direction, but what worries me is that if I do a time out, and then I pin the head that I might, because it’s very easy to go left, right, disorientation…. So I like them* [patients] *completely positioned and then we do timeout.* (Int 28, Surgeon)
	Being inclusive and patient-centred	• *I felt as a patient I was included and so, nobody was trying to conceal anything from me. The nurse in both cases was saying, “Ok, see the next question is” and so, you know, they will ask you about, you know, previous history or difficulties……*(Int 7, Healthcare Consumer) • *If the patient is awake and is having blocks of local anaesthetic and they say “time-out”, I say to the patient “Just listen. If any information about you or what they are saying in the next two minutes is wrong, say something”.* (Int 13, RN)
Working independently of others	Working in silos	• *We don’t work very well as a team. We have our two separate teams but we’re not a whole team. We’ve got two pods. An anaesthetic pod and a surgical pod.* (Int 4, 5 RNs) • *It’s “I have my job. You have your job” and I’m so like, why can’t we just help each other out? It seriously takes two seconds. You come in to the anaesthetics bay, you ask the patient usually five questions – name, date of birth, check the UR number, what are they having done, you check the consent form, you ask them if they have got any metal and when they last ate.* (Int 13, RN)
	Being task focussed	• *Fasting status is more of an anaesthetic nurse concern, but as a scrub or a scout I don’t… probably, when I’m doing the second and third components of the check, I probably don’t think “oh when did they last eat”. I’m concerned about that if I’m in a different role, but when I’m just a scrub scout in the theatre, I probably don’t….* (Int 1, RN) • *The anaesthetic nurse does the sign in bit by themselves. We usually go in and do our own little bit but it’s not the way that it’s meant to be done but it’s getting done and the sign out is just pointless all together.* (Int 4, 5 RNs)
	Being discipline-centric	• *But it’s the communication between the surgical team and the anaesthetic team is probably something that needs to be improved on. I guess it’s because we’re focused on two completely different aspects of this patient. One is focused solely on the airway and that they stay alive, and the surgical team are just… purely to get them in and out.* (Int 4 5 RNs) • *I don’t think they really pay attention to it* [anaesthetic checklist items]. *They [scrub nurses] pay attention to their own check, despite it being very important and a lot of it in their check as well.”* (Int 15, RN anaesthetics)
	Leading the process	• *I work both publicly and privately and somebody will call out the final check just before knife to skin. That’s what we would call timeout and I think that’s probably where I see my role, certainly as the anaesthetist, is often leading the timeout, certainly actively participating.* (Int 8, Anaesthetist) • *What I try to do is get everybody together, I try to get the wardsman and the theatre assistant, because they need to know the positioning of the patient, and if they don’t know ahead of time what the positioning of the patient is, then how can they do it?* (Int 9, Anaesthetist)
	Working in isolation	• *An example, this back bit with the sign-in bit, the scrub scout always comes to the anaesthetic nurse and says you haven’t ticked your bit. It’s like, did you watch the DVD? It doesn’t say that’s the anaesthetic nurse bit.* (Int 20, RN cardiac) • *The reality is, the anaesthetic nurse will fill this bit in, and she* [sic] *will leave blank the prosthesis and the essential imaging, and when the scrub scout … And then we leave those blank, and then the scrub scout does her* [sic] *little check, and she* [sic] *ticks those bits.* (Int 29, 5 RNs)
Communicating checks with others	Making sure, double checking	• *I’ll read the identification on the patient and get one of the nurses to ensure their consent, so they’ll double-check it and will ensure their consent on the site and obviously I will make sure that the surgeon is in the room at the time and I won’t let the nurses do it without ensuring that the surgeon is in the room.* (Int 8, Anaesthetist) • *And sometimes, the anaesthetic nurse will just pop the head in, in the theatre and say, “Have you got all the prosthesis?” You say, “Yeah.” Because you’re also opening and getting ready. So they just come in and ask …. And, and I’ll often check the screen to make sure that that patients’ scans are on the screen.* (Int 29, RNs x 5)
	Verbalising information	• *We should just verbalise it more, really. We’ve probably always done it. You know, you always check if your specimens are labelled, the equipment was okay, it’s what you always do but this is just a document that says you’ve done it. I think we just don’t verbalise that we’ve done it because it’s unsaid.*…(Int 2 8 RN) • *And then the time out, that’s done with everyone, we also incorporate stuff from the sign in, because we discuss antibiotics at that stage. Because it’s there in there, but we discuss it before the operation.* (Int 3, 8 Anaesthetic RNs)
	Handing over	• *But it all goes back to being handed over at the beginning. It always comes back to what is on it at the beginning when we’re passed on the information. I’ve had twice now, children come from the ward with no parent and no nurse to handover.* (Int 3, 8 Anaesthetic RNs) • *It’s usually… the anaesthetist. But we also get a handover from the surgical nurse who comes in and tells us what surgery they’ve* [patient] *had, perhaps what position they were in. If there were any problems with that, if they’ve had local anaesthetic, if they’ve had drains, dressings. Whether they were bleeding… Anything that might be relevant.* (Int 6, 3 PACU RNs)
	Asking meaningful questions	• *But those who are just used to doing tick and flick and not asking the deep meaningful questions, struggle when the meaningful questions are first asked…… (Int 9, Anaesthetist)* • *The best question on that safety checklist is implants or prosthetics. I ask patients “have you got any implants or prosthetics in your body?” They look at you like, “what’s that?” “Anything like metal work, pins, plates, screws, stents?” This is how I word it, “anything in your body you weren’t born with?” I suppose implants; people think breast implants and prosthetics, maybe limbs or something…. But it’s funny, as time goes on you figure out different ways of wording questions. (Int 14, RN)*

### Using the checklist

The category, *using the checklist* encompassed behaviours based on **checking processes** that occurred during the three checklist phases. Interview participants described how using the checklist enabled them to identify deficits and/or discrepancies in important patient information, thus minimizing or preventing potential errors or mistakes. Having multiple opportunities to ‘check and recheck’ information was considered imperative to keeping the patient safe and these repeated checks created “*layers of safety*” (Int 28, Surgeon). Some participants however warned that using the surgical safety checklist as a ‘tick-n-flick’ exercise may have the unintended effect of leading to complacency because team members were merely ‘going through the motions’ without listening to, or thinking about the information being collected. Surgical team members believed that ‘modifying and adapting’ the checklist enabled them to tailor their care to the particular needs of the patient as well as reflect the nuances of different surgical specialities. However some participants believed that the ability to modify the checklist or checking process had to be carefully balanced with the need to standardize the items covered in the checks to ensure that important information was communicated and was not lost or omitted during handover exchanges. Another component to checklist use was about ‘being inclusive and patient-centred’—to ensure that the patient, where possible was given the opportunity to participate in the conversation around their details and to speak up when the information was inaccurate. From a patient’s perspective, healthcare consumer interviewees believed that the conversation with the patient around the checklist items minimized the “*distance between you* [as patient] *and the healthcare professional*” (Int 7, Healthcare Consumer).

### Working independently

The category, *working independently* illustrated **team attributes** that shaped the ways surgical teams coordinated checklist activities, and which was largely, defined by their roles, and the associated task-related workflows. The majority of clinical participants believed that physicians should ‘take ownership’ and ‘lead the process’ of checking, particularly during the *time out* phase. While they did not always lead *timeout*, physicians advocated strongly that they should coordinate the *timeout* check as they ultimately had responsibility for the patient. In relation to overall accountability, this statement was typical of sentiments held by surgeon participants, “*I'm the one who takes the absolute risk*” (Int 18, Surgeon). During the observational audit, anaesthetists and surgeons were observed to initiate 20 and 40 % of *time out* checks respectively. The influence of ‘being task focussed’, ‘working in isolation’ and ‘working in silos’ was obvious in the way team members emphasized discipline-specific information and the limited coordination in checks conducted as a collective. Throughout the audit period, we observed that anaesthetic and scrub/scout nurses appeared to be automatically allocated different sections of the checklist. For example, during *sign in*, anaesthetic and scrub/scout nurses were regularly observed performing and documenting (ticking) their checks independently of each other, although these checks were part of the first phase of checks. These observations were confirmed in the interview data. The notion of role demarcation was again reinforced in ‘being discipline-centric’ as team members checked off particular checklist items based on their different discipline orientations.

### Communicating checks with others

The category, *communicating checks with others* highlighted the **communication strategies** used during the checking process. Strategies such as ‘making sure, double checking’, and ‘verbalizing information’ enabled team members to develop a shared mental model, ensuring that everyone was on the same page. The various checks that occurred as part of the preoperative and SSC permitted team members to reconfirm information at different junctures to circumvent or avert potential errors, inconsistencies or omissions in relation to consent, procedure/laterality/proximity, imaging, or allergies. Likewise, the deliberate act of verbalizing information offered participants the opportunity to engage in a dialogue, especially during time out. Clinical participants advocated strongly that the *time out* check in particular, needed to be more “patient centric” and done while the patient was still awake and able to contribute to the conversation, rather than after anaesthetic induction. Nurse participants considered the patient ‘hand over’ process during *sign in* and patient transfer from the operating room to the post anaesthetic recovery unit (PACU), critical to providing seamless, safe care. However when the checklist was carried out as a *“tick box”* process, team members’ ability to ‘ask meaningful questions’ and elicit relevant information from patients and other team members was reduced.

**Fig. 1:**
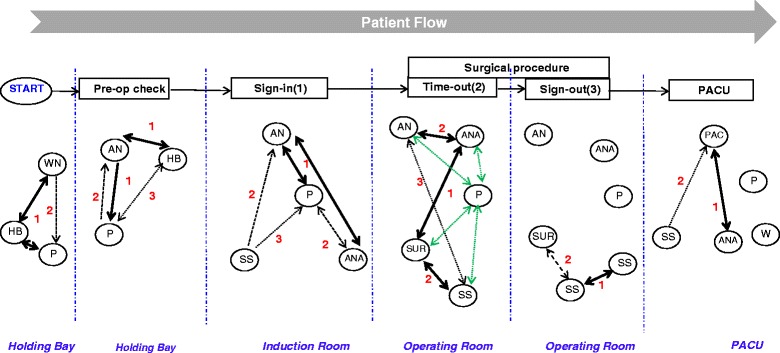
Personnel involved in team communications during phases of the Preoperative and Surgical Safety Checklists (elective procedures). Note: P= Patient; WN= Ward Nurse; HB=Holding Bay Nurse; AN=Anaesthetic Nurse; ANA=Anaesthetist; S-S=Scrub-Scout Nurse; SUR=Surgeon; PACU=Post-Anaesthetic Care Unit; PAC=PACU Nurse; W=Wardsman;  = One-way exchange;  = Two-way exchange;  = Infrequent exchange;  = Exchange semi-frequent;  = Frequent exchange;  = Patient awake & included in ‘timeout’ checks

During field work, we mapped team communications in relation to who was present and who participated in each phase of the checking process, and the proportion of checklist items that were verified and confirmed. Figure 1 illustrates the personnel involved in information exchanges at each juncture of the preoperative and surgical safety checklists. Throughout *sign in* and *sign out* phases, individual team members performed checks and communicated these as the need arose. Sometimes when the scrub and scout nurses were busy setting up for the next procedure, the anaesthetic nurse who had performed *sign in* would enter the OR to handover information to scrub/scout nurses about *patient consent, procedure*, and *allergies*. Anaesthetists were not directly involved in the *sign in* process, performed their own preoperative checks, asking patients information about medical history, allergies, and medications. Overall, total item completion of *sign in* items was 52.1 %. Anaesthetic nurses were primarily involved in *sign in* while only the scrub and scout nurses performed *sign out*. We did not observe participation by any other team members during *sign out*. Notably, *sign in* and *sign out* were the most vulnerable checklist phases because of the increased risk for information loss or omission.

Team participation was at its greatest during the ‘time out’ phase. That is, all team members were consistently present and simultaneously exchanged clinical information. During observations, *timeout* checklist items pertaining to *patient identity* and *procedure* were always stated and verified as a collective, while *site/side* was checked and confirmed in 60 % of cases. Confirmation of checklist items; *critical and non-routine steps* and *patient-specific concerns* was observed in 40 and 50 % of cases respectively. Significantly, verbal confirmation of *equipment availability* and *sterility* by the team was not observed.

## Discussion

In this study we have identified and described the factors that drive team participation in safety checks. To facilitate description, we mapped out team member participation around the frequency/regularity of communication exchanges for each phase of the checklist. This study expands on the growing body of knowledge on this important field and identifies potential points of vulnerability across the checking process. The drivers of surgical safety checklist participation, while distinct, are interrelated: Team participation depends on a confluence of interlinked factors; namely, *team attributes, communication strategies* and *checking processes*.

### Team attributes

Our findings highlight that team attributes reflect independence on an individual level versus interdependence on a team level. Professional independence requires that team members possess a tacit knowledge of regimens of care, therefore permitting members to perform their work tasks in a synchronized, unified manner [38]. Clearly being self-directed and working independently may improve teamwork. Yet, a significant limitation to professional independence is the siloing that comes as a result of a focus on professional role, which constrains opportunities for deliberate, open discussion among team members as a collective [39]. Study participants’ expectations that team members would naturally perform their sections of the checklist, with little, if any communication about how this would or should be done reinforced a task-oriented approach. In some situations, a task-orientated approach is required (e.g., patient intubation, micro-surgery) but without explicit communication about performing the checks, vital information may be missed and/or not passed on. Professional identity is accentuated by the “tribal affiliations” of members and the contrast between physicians’ and nurses’ traditional role responsibilities and teamwork behaviours in surgery [38, 40–42]. The disciplinary focus of team members necessarily determines what information is communicated and when, and with whom it is communicated [21]. Participants in our study described the importance of having a designated leader for each phase of the checklist, and resolutely believed that physicians should lead *time out*. Findings from a recent realist synthesis of 35 studies found that ‘professional identity’ characterized checklist implementation, which is discipline-specific and more likely successful when physicians are actively leading and participating in the process [18]. **Team attributes** is the biggest factor in checklist participation and drives the communication strategies used when performing the phases of the checks.

### Communication strategies

Our observational and interview findings suggest that checklist communications especially during the *sign in* and *sign out* phases occurred with individuals serendipitously, rather than actively or deliberately. Minimal communication in surgery is perceived as acceptable in OR culture—ostensibly effective because it demonstrates clinical competence which is considered the epitome of safety [43, 44]. However, minimal or irregular team communications can also conceal inconsistencies or omissions in information [22, 25, 45]. Communicating task-related information also enables greater situation awareness [42], and thus allows team members to build shared mental models. From a safety perspective, checklists are regarded as a defence against adverse events [46], and when handover is incomplete, the risk of error increases. Similarly, when checks are performed as a tick box activity, the risk of failure to detect errors increases [47]. In our study, participants’ perceptions of risk seemed only apply to active failures and not latent conditions. Notably, physicians actively participated only in the *timeout* phase of the checklist as they believed that this was the phase where errors were most likely to be detected, a point highlighted in previous work [48, 49]. Nevertheless, as study participants identified, communications around the checks must be meaningful and the information sought, relevant and useful. That is, team members must ask pertinent questions to attain accurate clinical information that will be used to inform clinical decision making.

### Checking processes

Our findings show that the *timeout* phase of the checklist was most valued by participants, and hence, had the highest level of team participation and completion rates. Most likely because *timeout* is the one time that the team whole is together with the patient. The *sign out* process was not a team-based activity, albeit that scrub and scout nurses performed checks at various time points during the procedure while multitasking as opposed to performing these checks during wound closure. Participants perceived *sign out* items as nebulous and unclear, and held disparate perceptions of when to initiate the *sign out* phase, likely because of conflicting individual workflows. Competing workflows consequently led to potential discrepancies or incomplete checks. That *sign in* and *sign out* checks were not confirmed and validated by the entire surgical team is largely driven by **team attributes** and **communication strategies**. Does it really matter that a team-based approach is not used for the *sign in* and *sign out* phases? Deliberate confirmation with oral validation of checklist items promotes closed loop communication and gives others the opportunity to ask questions and clarify concerns [50, 51]. Ideally involving the patient in confirmation and validation of information during the *sign in* and *time out* phases also adds another layer of safety. While errors in specimen labelling were not observed during fieldwork, several nurse participants reported mislabelling errors that were picked up during the *sign out* phase. Implementing behaviour change interventions aimed at educating and reminding staff about performing the safety checks, i.e., *who does what, when and how* may partially address gaps in participation. However, the broader issue around **team attributes** requires behaviour change interventions that address professional culture and systemic issues such as workflow.

### Strengths and limitations

Despite the use of a single hospital site; we spent extensive time interviewing participants and stakeholders including patients, thus allowing diverse perspectives. Such diversity may allow conceptual transference of findings to other similar settings. Twelve months prior to the commencement of this study, hospital staff had relocated to a larger facility, where the geographic layout was very different. This may have impacted on team communications because of the size of the facility. Finally, the dissimilar interview methods may have given rise to different group dynamics but similar issues were discussed and data saturation achieved.

## Conclusions

While checklists are powerful tools to standardize key work processes, they are often viewed as a ‘simple’ solution to addressing consistency in team communications. Further, the use of checklists is never formally taught in a structured manner, making embedding checklists in clinical practice even more challenging. Adopting a whole-of-team approach to participation in surgical safety checks is far more complex when considering the factors that drive participation. Implementing checklists in surgery involves complex social interactions between surgeons, anaesthetists and nurses with the expectation of cooperation. We recommend implementation strategies that specifically target team communication processes and systemic issues such as workflow.

## References

[1] Kohn L, Corrigan J, Donaldson M (2000). To err is human: Building a safer health system.

[2] Thomas E, Studdert D, Burstin H, Orav E, Zeena T, Williams E (2000). Incidence and types of adverse events and negligent care in Utah and Colorado. Med Care.

[3] Wilson R, Runciman W, Gibbard R, Harrison B, Newby L, Hamilton J (1995). The quality in Australian health care study. Med J Aust.

[4] de Vries E, Ramrattan M, Smorenburg S, Gouma D, Boermeester M (2008). The incidence and nature of in-hospital adverse events: a systematic review. Qual Saf Health Care.

[5] Kable A, Gibberd R, Spigelman A (2008). Predictors of adverse events in surgical admissions in Australia. Int J Qual Health Care.

[6] Gawande A, Zinner M, Studdert D, Brennan T (2003). Analysis of errors reported by surgeons at three teaching hospitals. Surgery.

[7] World Health Organization (2008). Impllementation of the Surgical Safety Checklist.

[8] Askarian M, Kouchak F, Palenik CJ (2011). Effect of surgical safety checklists on postoperative morbidity and mortality rates, Shiraz, Faghihy Hospital, a 1-year study. Qual Manag Health Care.

[9] Berrisford R, Wilson I, Davidge M, Sanders D (2012). Surgical time out checklist with debriefing and multidisciplinary feedback improves venous thromboembolism prophylaxis in thoracic surgery: a prospective audit. J Cardiothorac Surg.

[10] Bliss LA, Ross-Richardson CB, Sanzari LJ, Shapiro DS, Lukianoff AE, Bernstein BA (2012). Thirty-day outcomes support implementation of a surgical safety checklist. J Am Coll Surg.

[11] Haynes A, Weiser TG, Berry W, Lipsitz S, Breizat A, Dellinger EP (2009). A surgical safety checklist to reduce morbidity and mortality in a global population. N Engl J Med.

[12] Kwok A, Funk L, Baltaga R, Lipsitz S, Merry A, Dziekan G (2012). Implementation of the World Health Organization surgical safety checklist, including introduction of pulse oximetry, in a resource-limited Setting. Ann Surg.

[13] Yuan CT, Walsh D, Tomarken JL, Rachelle A, Shakpeh J, Bradley EH (2012). Incorporating the World Health Organization surgical safety checklist into practice at two hospitals in Liberia. Jt Comm J Qual Patient Saf.

[14] Borchard A, Schwappach D, Barbir A, Bezzola P (2012). A systematic review of the effectiveness, compliance, and critical factors for implementation of safety checklists in surgery. Ann Surg.

[15] Bergs J, Hellings J, Cleemput I, Zurel O, De Troyer V, Van Hiel M (2014). Systematic review and meta-analysis of the effect of the World Health Organization surgical safety checklist on postoperative complications. Br J Surg.

[16] Gillespie BM, Chaboyer W, Thalib L, Fairweather N, Slater K (2014). Effect of using a safety checklist in surgery on patient complications: A systematic review and meta-analysis. Anaesthesiology.

[17] Bosk C, Dixon-Woods M, Pronovost PJ (2009). The art of medicine reality check for checklists. N Engl J Med.

[18] Gillespie BM, Marshall A (2015). Implementation of safety checklists in surgery: a realist synthesis of evidence. Implement Sci.

[19] Fourcade A, Blache JL, Grenier C, Bourgain JL, Minvielle E (2012). Barriers to staff adoption of a surgical safety checklist. BMJ Qual Saf.

[20] Russ S, Sevdalis N, Moorthy K, Mayer E, Rout S, Caris J, et al. A Qualitative Evaluation of the Barriers and Facilitators Toward Implementation of the WHO Surgical Safety Checklist Across Hospitals in England Lessons From the “Surgical Checklist Implementation Project”. Ann Surg. 2015 Jan;261(1):81-91. doi: 10.1097/SLA.0000000000000793. 10.1097/SLA.0000000000000793 25072435

[21] Gillespie BM, Chaboyer W, Wallis M, Fenwick C (2010). Why isn’t time out being implemented? An exploratory study. Qual Saf Health Care.

[22] Biffl W, Gallagher A, Pieracci F, Berumen C (2015). Suboptimal compliance with surgical safety checklists in Colorado: A prospective observational study reveals differences between surgical specialties. Patient Saf Surg.

[23] Truran P, Critchley RJ, Gilliam A (2011). Does using the WHO surgical checklist improve compliance to venous thromboembolism prophylaxis guidelines?. Surgeon.

[24] Levy S, Senter C, Hawkins R, Zhao J, Doody K, Kao L (2012). Implementing a surgical checklist: More than checking a box. Surgery.

[25] García-París J, Coheña-Jiménez M, Montaño-Jiménez P, Córdoba-Fernández A (2015). Implementation of the WHO “Safe Surgery Saves Lives” checklist in a podiatric surgery unit in Spain: a single-center retrospective observational study. Patient Saf Surg.

[26] Sparkes D, Rylah B (2010). The World Health Organization surgical safety checklist. Br J Hosp Med (Lond).

[27] Kasatpibal N1, Senaratana W, Chitreecheur J, Chotirosniramit N, Pakvipas P, Junthasopeepun P (2012). Implementation of the World Health Organization surgical safety checklist at a university hospital in Thailand. Surg Infect (Larchmt).

[28] Vogus T, Sutcliffe K (2007). The safety organizing scale: development and validation of a behavioral measure of safety culture in hospital nursing units. Med Care.

[29] Nwosu A (2015). The horror of wrong-site surgery continues: report of two cases in a regional trauma centre in Nigeria. Patient Saf Surg.

[30] Kitto S, Chesters J, Grbich C (2008). Quality in qualitative research: Criteria for authors and assessors in the submission and assessment of qualitative research articles for the Medical Journal of Australia. Med J Aust.

[31] Klueger R (1994). Focus groups: A practical guide for applied research.

[32] Michie S, Johnson M, Abraham C, Barker D, Walker A, Group obotPT (2005). Making psychological theory useful for implementing evidence based practice: a consensus approach. Qual Saf Health Care.

[33] Guba E, Lincoln Y, Denzin N, Lincoln Y (1994). Competing paradigms in qualitative research. Handbook of qualitative research.

[34] DeSantis L, Ugarriza D (2000). The concept fo theme as used in qualitative nursing research. West J Nurs Res.

[35] Strauss A, Corbin J (1998). Basics of qualitative research. Grounded theory procedures and techniques.

[36] Shih F-S (1998). Triangulation in nursing research: Issues of conceptual clarity and purpose. J Adv Nurs.

[37] Straus S, Tetroe J, Graham I (2011). Knowledge translation is the use of knowledge in health care decision making. J Clin Epidemiol.

[38] Gillespie BM, Chaboyer W, Longbottom P, Wallis M (2010). The impact of organisational and individual factors on team communication in surgery: A qualitative study. Int J Nurs Stud.

[39] Lingard L, Regehr G, Cartmill C, Orser B, Espin S, Bohnen J, et al. Evaluation of a preoperative team briefing: a new communication routine results in improved clinical practice. BMJ Qual Saf. 2010. doi: 10.1136/bmjqs.2009.032326. 10.1136/bmjqs.2009.032326 21303767

[40] Bleakley A, Boyden J, Hobbs A, Walsh L, Allard J (2006). Improving teamwork climate in operating theatres: The shift from multiprofesisonalism to interprofessionalism. J Interprof Care.

[41] Sexton B, Makary M, Tersigni A, Pryor D, Hendich A, Thomas E (2006). Teamwork in the operating room. Aneasthesiology.

[42] Lingard L, Regehr G, Orser B, Reznick R, Baker R, Doran D (2008). Evaluation of a Pre-operative checklist and team briefing among surgeons, nurses, and anaesthesiologists to reduce failures in communication. Arch Surg.

[43] Lingard L, Epsin S, Rubin B, Whyte S, Colmenares M, Baker G (2005). Getting teams to talk: development and pilot implementation of a checklist to promote interprofessional communication in the OR. Qual Saf Health Care.

[44] Gillespie BM, Gwinner K, Fairweather N, Chaboyer W (2013). Building shared situational awareness in surgery through distributed dialog. J Multidiscip Healthcare.

[45] Lingard L, Whyte S, Espin S, Baker R, Orser B, Doran D (2006). Towards safer interprofesional communication: Constructing a model of “utility” from preoperatvie team briefings. J Interprof Care.

[46] Reason J (2005). Safety in the operating theatre - Part 2: Human error and organisational failure. Qual Saf Health Care.

[47] Cullati S, Licker MJ, Francis P, Degiorgi A, Bezzola P, Courvoisier DS (2014). Implementation of the surgical safety checklist in Switzerland and perceptions of its benefits: cross-sectional survey. PLoS One.

[48] Thomassen Ø, BrattebøJon G, Heltne K, Søfteland E, Espeland A (2010). Checklists in the operating room: Help or hurdle? A qualitative study on health workers’ experiences. BMC Health Serv Res.

[49] Conley D, Singer S, Edmondson L, Berry WR, Gawande AA (2011). Effective surgical safety checklist implementation. J Am Coll Surg.

[50] Bell R, Pontin L (2010). How implementing the surgical safety checklist improved staff teamwork in theatre. Nurs Times.

[51] Sutcliffe KM, Lewton E, Rosenthal MM (2004). Communication failures: An insidious contributor to medical mishaps. Acad Med.

